# The Critical Shoulder Angle: A Significant Radiological Measure in Rotator Cuff vs. Glenohumeral Osteoarthritis in Chilean Patients—A Descriptive Cross-Sectional Study

**DOI:** 10.3390/jcm13123408

**Published:** 2024-06-11

**Authors:** Walter Rojas, Pablo Vargas, Guillermo Droppelmann, Carlos Jorquera, Katherine Stöwhas, Alejandro Godoy, Nicolás García

**Affiliations:** 1 Clínica MEDS, Santiago 7691236, Chile; walter.rojas@meds.cl (W.R.); pablo.vargas@meds.cl (P.V.); katherine.stowhas@meds.cl (K.S.); nicolas.garciaa@meds.cl (N.G.); 2 Escuela de Medicina, Facultad de Medicina, Universidad de Valparaíso, Valparaíso 2540064, Chile; agodoyg@gmail.com; 3 Harvard T.H. Chan School of Public Health, Boston, MA 02115, USA; 4 Facultad de Ciencias, Escuela de Nutrición y Dietética, Universidad Mayor, Santiago 8580745, Chile; carlos.jorquera@mayor.cl; 5 Facultad de Medicina, Escuela de Kinesiología, Universidad Finis Terrae, Santiago 7501014, Chile

**Keywords:** critical shoulder angle, glenohumeral osteoarthritis, rotator cuff injury, shoulder pain

## Abstract

**Background:** Shoulder pain is one of the most important musculoskeletal conditions affecting the upper extremities. Glenohumeral osteoarthritis (GHOA) and rotator cuff injuries (RCIs) are notable for their high prevalence. The critical shoulder angle (CSA) is a significant radiological measure for determining the diagnosis and progression of patients with these conditions. Although there are reports in the international literature about this measure, in our country, guideline values considering these two pathologies are unknown. **Objective:** Our objective was to assess patients diagnosed with GHOA and RCI using an AP X-ray view and the CSA. **Methods:** To conduct this, we identified differences between sexes and age categories. Fifty-nine adult patients with GHOA and RCI were included. CSA grades varied depending on the age category and type of injury evaluated. **Results:** Significant differences between the age ranges of 40 and 54 (*p* = 0.05), 55–69 (*p* = 0.001), and 70–84 (*p* = 0.017) were observed. **Conclusions:** Patients with RCI tended to be younger and have a higher CSA compared to those with GHOA. It is important to have more normative values and to continue monitoring the critical shoulder angle in these patients.

## 1. Introduction

Shoulder pain is one of the most disabling musculoskeletal conditions, making it one of the top three most prevalent pathologies of this system after lower back and knee pain [1]. It affects a considerable proportion of the population worldwide on a daily, yearly, and lifelong basis [1]. Although clinical manifestations play key roles in the initial diagnosis and management decisions, radiological evaluation is also one of the most important aspects of diagnosis and monitoring the evolution of patients with those diseases [2]. A variety of radiographic views have been described in the literature for the initial imaging of non-acute shoulder pain [3], especially in chronic shoulder pain, including glenohumeral osteoarthritis (GHOA) [4] and rotator cuff injuries (RCI) [5]. In particular, the critical shoulder angle (CSA) is formed between the glenoid fossa plane and a line drawn from the inferior edge of the glenoid to the lateral edge of the acromion on a true anteroposterior (Grashey) shoulder radiograph [6]. There is evidence that a wider CSA is typically associated with full-thickness rotator cuff tears, whereas a smaller CSA is generally associated with GHOA [7,8]. However, there is no evidence to support the reproducibility and accuracy of CSA values when measured using radiographs in Chilean patients with GHOA and RCI. This highlights the importance of establishing the relationship between the CSA and shoulder pathologies such as RCI and GHOA. It underscores the crucial need for reliable, effective, and affordable evaluation and diagnostic tools that are accessible to a wide range of people [8]. Such tools could significantly aid in predicting shoulder pathologies in patients with shoulder complaints in primary care, enabling the early detection of severe shoulder pathologies and the timely application of appropriate treatment [9].

In this regard, osteoarthritis is the world’s most common joint disease, and there is currently no cure. In particular, GHOA alone accounts for an estimated 5–17% of patients with shoulder pain and has proven to be a major contributor to shoulder joint pain and dysfunction in the elderly [10]. However, recent publications affirm that the prevalence of radiological shoulder OA has been estimated to be as high as 16–20% in the middle-aged and elderly population [11]. Thus, early clinical factors for diagnosis of this condition are of paramount importance [12]. On the other hand, RCI, another high source of shoulder pain, runs the full spectrum from injury to tendinopathy to partial tears and finally complete tears. Age also plays a significant role. Injuries range from 9.7% in those 20 years and younger, increasing to 62% in patients 80 years and older (whether or not they have symptoms) [13]. Therefore, the origin of the pain is uncertain and influenced by a variety of factors. While clinical manifestations play key roles in initial diagnosis and management decisions, the radiological evaluation is also crucial [14], and a recent meta-analysis determined that the main factors associated with the RCI were the following demographics: older age, greater body mass index, smoking, and dominant arm [11,15].

In this case, ultrasound and magnetic resonance imaging (MRI) are high-resolution imaging techniques that allow for the complete assessment of shoulder conditions [16,17]. In particular, when the medical team needs to further evaluate unusual diagnostic suspicions, having high spatial resolution and multiplanar imaging capabilities to evaluate the shoulder joint is necessary [18]. Several radiographic views are recommended in the literature for the initial imaging of chronic shoulder pain, including conditions like GHOA and RCI. The imaging protocol often begins with an anteroposterior (AP) view [19], which is useful for assessing disease progression [20]. This examination is based on the fact that GHOA, the product of a traumatic or degenerative tear of the rotator cuff, shows three characteristic changes. These are rotator cuff insufficiency, cranial migration of the humeral head, and subsequent radiographic degenerative changes. Radiographs usually show the bony erosion of the superior glenoid, resulting in acetabularization of the coracoacromial arch and the rounding of greater humeral tuberosity [11]. A recent meta-analysis determined that three factors are determining factors in the behavior of these injuries. The first is a greater acromion index, a smaller glenoid version angle, and a greater critical shoulder angle (CSA) [21]. Primary glenohumeral osteoarthritis is characterized by a notably smaller cross-sectional area, whereas degenerative rotator cuff tears are linked to a significantly larger cross-sectional area compared to asymptomatic shoulders. To date, multiple studies have addressed the topic of CSA [22,23,24,25,26,27]. However, the current literature offers both supporting and conflicting evidence regarding the relationships between CSA, shoulder disease, and clinical treatment outcomes. These discrepancies may be attributed to the absence of standardized radiographic methods and measurement inaccuracies in CSA measurement. Several studies conducted worldwide have examined various populations, including Brazil [28], Japan [29], and the East Asian population [30], among others. However, in Chile, only one publication has been reported, focusing on acromial morphology rather than CSA [31]. This makes it essential to identify CSA values in the Chilean population affected by GHOA or RCI and categorize them according to sex and age categories.

The objective of the present study is to identify CSA in Chilean patients with GHOA and RCI by sex and age following international experiences.

## 2. Materials and Methods

### 2.1. Ethics Statement

The latest version of the Declaration of Helsinki and Chilean scientific legislation guided the conduct of this study. Approval for this study was obtained from the “Comité de Ética Científico Adulto del Servicio Metropolitano Oriente (SSMO) de la Ciudad de Santiago de Chile” (IRB). As this was a retrospective study, informed consent was not required by the SSMO Ethical Committee (IRB). The SSMO Ethical Committee approved the project in June 2021.

### 2.2. Study Design

This research was designed as a retrospective descriptive cross-sectional study conducted at two sites, following the guidelines outlined in the Strengthening the Reporting of Observational Studies in Epidemiology (STROBE) checklist (https://www.strobe-statement.org/, accessed on 30 May 2024). Patient records were exclusively obtained from Rx examinations conducted at the MEDS Clinic and San José Hospital, both located in Santiago, Región Metropolitana, Chile. The study commenced in July 2021.

### 2.3. Participants

The team of researchers gathered the necessary information from patients diagnosed with GHOA and RCI from both sexes and those over 18 years of age. All the radiograph examinations analyzed belonged to patients who were treated at the mentioned healthcare centers. The MEDS Clinic is a healthcare provider in Chile’s private healthcare system. Meanwhile, the San José Hospital is a care center belonging to the public healthcare system. Both institutions are defined as high-complexity healthcare facilities

### 2.4. Sample Size and Recruitment

A convenience sampling method was employed. The estimation of the number of subjects to be evaluated was conducted using predefined criteria based on previous Latin-American publications. All patients diagnosed with the aforementioned conditions at the specified healthcare institutions were included in the study. Demographic information was extracted from their clinical records, and anteroposterior X-rays of the shoulder were obtained.

### 2.5. Selection Criteria

The inclusion criteria consisted of patients requiring anatomical shoulder arthroplasty due to supraspinatus or supraspinatus plus infraspinatus tendon ruptures, as confirmed by MRI. Additionally, patients were required to undergo a shoulder X-ray with an anteroposterior view within 6 months prior to surgery or surgical indication and to have a Samilson type 3 classification stage of primary glenohumeral osteoarthritis.

Exclusion criteria included previous shoulder surgery, rotator cuff tears deemed irreparable, tears of the subscapularis and/or teres minor, arthropathy resulting from a Hamada rotator cuff tear type 1 or more, glenoid and/or humeral dysplasia, previous joint infection, and autoimmune arthropathy.

### 2.6. Personal Variables

Sociodemographic variables, such as age, sex, dominance, side of injury, and type of shoulder condition, were identified.

### 2.7. Assessment of Critical Shoulder Angle

**Figure 1:**
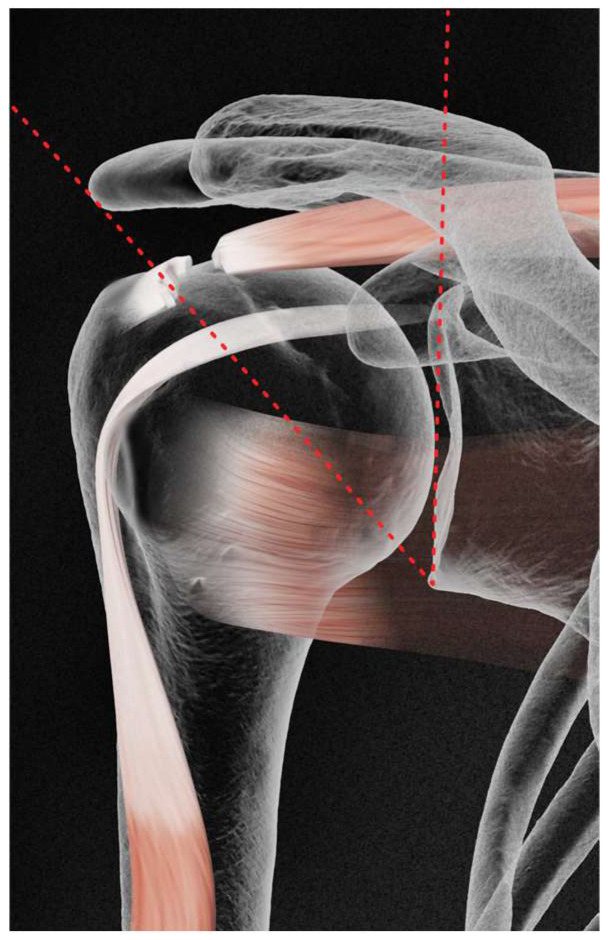
CSA measure in RCI.

**Figure 2:**
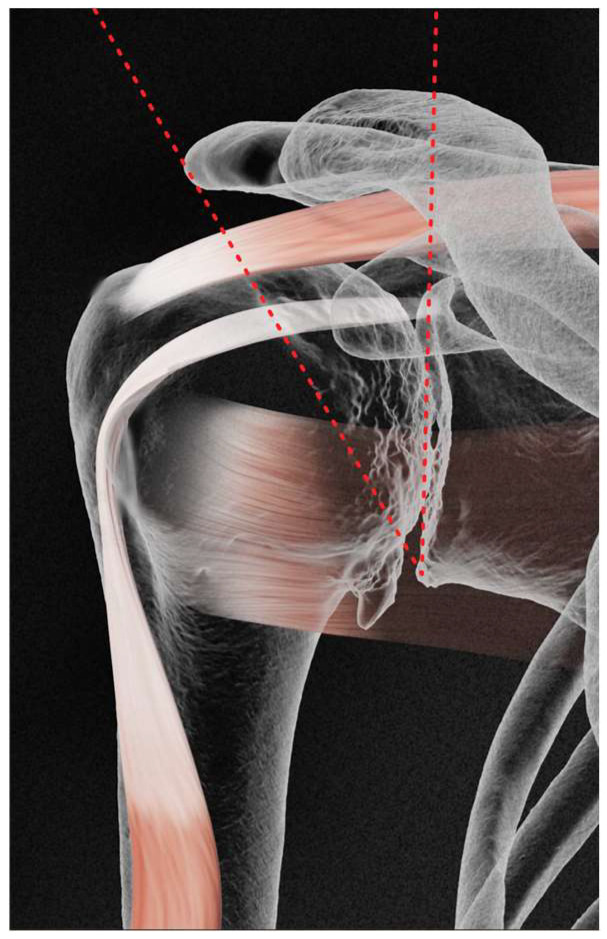
CSA measure in GHOA.

All examined patients who met the selection criteria had their CSA measured. The CSA corresponded to the angle obtained by the conjunction between the following two lines: the first line is drawn by joining the superior and inferior bony margins of the glenoid; the second line joins the inferior bony margin of the glenoid to the most lateral border of the acromion. Figure 1 and Figure 2 demonstrate this.

A board-certified radiologist surgeon with a subspecialty in musculoskeletal injuries and over 15 years of experience performed the radiographic measurements.

The aforementioned observer randomly recorded and analyzed all radiographs, ensuring no personal information was present. The CSA was digitally measured using the Phillips Radiology Information System.

### 2.8. Statistical Analyses

Descriptive and inferential statistics were applied. The distribution of the frequency of the variables of age, sex, dominance, and affected side according to pathology was described. Furthermore, the percentage frequency of the CSA ranges by intervals depending on the shoulder condition was estimated. Finally, differences were calculated according to the age range and condition. Normality assumptions were assessed using the Shapiro–Wilk test. To check for differences in the variables, the *t*-test, Mann–Whitney’s, and Fisher’s exact tests were used.

To assess the reliability of the CSA measures in this study, we computed the two-way random effect intraclass correlation coefficient (ICC2,k) using absolute agreement. Additionally, we calculated the 95% confidence intervals (CIs) [32]. For this study, we interpreted the ICC values according to the following criteria: values less than 0.5, between 0.5 and 0.75, between 0.75 and 0.9, and greater than 0.90 were indicative of poor, moderate, good, and excellent reliability, respectively [33].

The level of statistical significance was set at *p* ≤ 0.05. All data were analyzed using IBM SPSS Statistics 28 (IBM Corp., Armonk, NY, USA) and the R statistical software (R version 4.3.1).

## 3. Results

### 3.1. Participants

**Figure 3:**
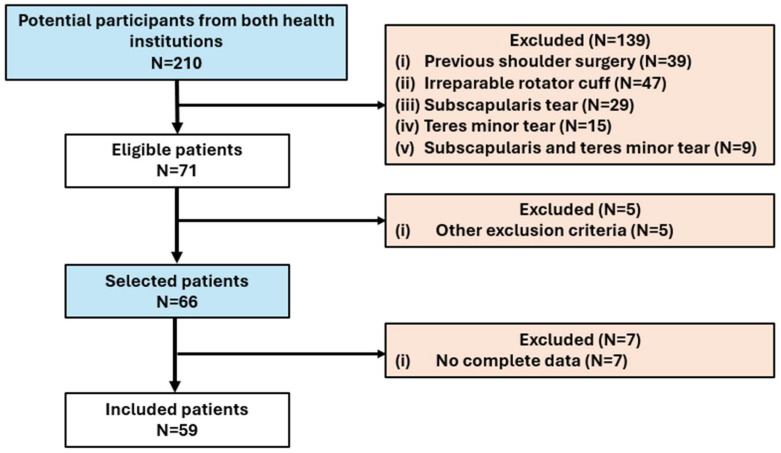
Participant flow.

Fifty-nine patients with shoulder pain underwent X-ray examinations with an anteroposterior view. Twenty-nine patients presented with glenohumeral osteoarthritis (GHOA), while the remainder received a diagnosis of rotator cuff injury (RCI). Of the total participants in the examination, 33 (55.9%) were female. The mean age was 66.85 (±9.3) years, with a significant difference between the sexes (*p*-value < 0.001). Figure 3 represents the flow of examination participants.

The evaluator calculated the average of various random measurements. The intraclass correlation coefficient between the evaluations obtained a value of 0.824 (95% CI: 0.742–0.885), indicating a high level of agreement in the evaluations performed by the specialist.

On the other hand, significant differences (*p* < 0.05) were observed between age and the dominant shoulder. However, no significant differences (*p* > 0.05) were observed between the sex and injury sides. Further information is available in Table 1.

### 3.2. CSA Results by Shoulder Condition

**Figure 4:**
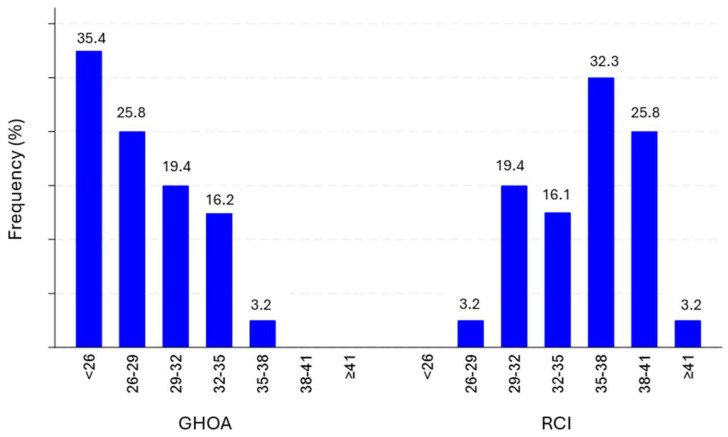
Distribution of CSA according to pathology.

The distribution of the GHOA variable was analyzed in a sample of 31 cases. The results demonstrate variability in the distribution of CSA angle ranges. The most frequent group fell within the CSA angle range of less than 26, comprising a total of 11 cases, which accounted for 35.4% of the total. The CSA angle ranged between 26 and 29 was followed, accounting for 8 cases (25.8%). Subsequently, the number of cases decreased progressively: 6 cases (19.4%) in the range between 29 and 32, 5 cases (16.2%) between 32 and 35, and 1 case (3.2%) between 35 and 38. No cases were recorded for the CSA angle ranges between 38 and 41, nor in groups of 41 or more. Additionally, the distribution of the variable was analyzed in a sample of cases. The results showed variability in the CSA angle intervals. The category recorded no cases with a value less than 26. One case (3.2%) fell within the interval between 26 and 29. The interval between 29 and 32 contained the majority of cases, specifically six (19.4%). Five cases (16.1%) fall within the range between 32 and 35. The interval between 35 and 38 exhibited the highest concentration of cases, accounting for ten cases (32.3%). The interval between 38 and 41 registered eight cases (25.8%). Finally, we found one case (3.2%) in the category with a value equal to or greater than 41. See Figure 4 for more detail.

### 3.3. CSA Results by Shoulder Condition and Age

The results suggest that the presence of GHOA and RCI varies according to the age group, with statistically significant differences observed, especially in the 40–54, 55–69, and 70–84 groups. The results show that the age group of 40 to 54 years had slightly lower statistical significance than the other groups. In the age group of 55 to 69 years, there was an average value of 26.5 for GHOA and 35.4 for RCI, with a statistically significant difference between both groups (*p* < 0.001) regarding RCI. In the age group of 70 to 84 years, the mean GHOA value was 30.3, and the mean RCI value was 35.6, with a statistically significant difference between both groups (*p* = 0.017), albeit less significant than in the 55 to 69 age group. The age group of 85 to 100 years displayed an average GHOA value of 27.6 and provided no RCI value. See Table 2 for more details.

## 4. Discussion

This study estimated one of the most commonly used radiological measures for shoulder injuries by orthopedic specialists. The CSA utilized enabled the identification of the shoulder joint’s health status as well as tracking disease progression in patients with GHOA and RCI [26]. Our research uncovered an initial approach to identifying values according to sex and different age categories in a sample of the population belonging to both a public and a private institution within the Chilean healthcare system. In particular, we observed no differences by sex, which aligns with recent international experiences [34], but we identified statistically significant differences by the age stratum according to pathology. These results are consistent with those reported by other authors [35], highlighting the importance of obtaining a successful registration.

In this sense, our results are consistent with other reports made in the literature where it is shown that 84% of patients with RCI had CSA values of >35°, while 93% of patients with GHOA demonstrated CSA values of <30° [36]. At the same time, previous studies have indicated that the highest prevalence of shoulder pain occurs in individuals under 65 years of age [37]. Our results are consistent with this assertion, but in the group of subjects presenting with RCI, the GHOA group is higher than the proposed range. Indeed, one could explore the hypothesis that a rotator cuff tear is a potential predictor of developing a GHOA-type injury in the short and medium term [38]. However, this is still controversial, as some studies claim that CSA does not change over time but rather remains stable [39].

An anteroposterior X-ray of the glenohumeral complex is crucial not only because it is cost-effective and aids doctors in assessing bone structures [40] but also because it helps to identify potential soft tissue injuries [41]. International reports suggest that patients with a high CSA and pain may potentially present unresolved soft tissue injuries, necessitating further studies to confirm this association [42]. Because the CSA is the main force acting on the glenohumeral joint and the rotator cuff, it might be important to consider it in both RCI and GHOA [8]. This is especially true for the supraspinatus tendon, partially explaining why it may rupture, particularly if the patient is experiencing pain [43]. Recently, a meta-analysis confirmed an association between high CSA indices and a higher risk of rotator cuff tear [14]. Previous studies have also confirmed that CSA measurements greater than 30 to 35 degrees pose a higher risk of presenting an RCI [36,44]. On the other hand, a study evaluating Caucasian patients found that the average CSA was 33.6 degrees, with no significant differences by sex but a trend towards an increase in the angle with aging [45]. However, the study did not link it to the development of pathologies like those evaluated in this article.

We believe that a more standardized assessment of the shoulder complex is critical, and the medical team should always include a radiographic image in the physical examination to monitor the health status of the shoulder joint [46]. We understand that initial findings could potentially lead to changes in both RCI and lesions, such as GHOA. The measurement of CSA, as well as its recording and follow-up over time, should always be present in the shoulder assessment, especially if the individual has a family history, comorbidities, or physical demands from sports or work [44,47].

Previous studies have only measured this angle using radiographs [45]. However, among the main strengths of our study, we emphasize that all of our patients had a radiographic image in the anteroposterior view as well as an MRI that confirmed both RCI and GHOA lesions, as suggested by some authors, to determine diagnostic accuracy more precisely [24]. Furthermore, we ensured that the measurements were as precise as possible by having a radiologist specializing in musculoskeletal dysfunctions with more than 15 years of experience conducting the measurement tests. This radiologist underwent concordance tests to estimate better values, which is a process not always reported as standardized in the literature [21]. Overall, we emphasize that half of the patients were part of our country’s public health system, while the other half belonged to the private health system. Despite improper measurements, some studies suggest potential differences among participants [48], potentially reducing gaps in the selected sample.

This article presents several limitations. Firstly, there is a small sample size, although it was sufficient to obtain differences between the selected categories; however, we suggest obtaining a larger and more representative sample of the population. This is because all our patients had an MRI scan, making it more complex in practice to access a sample with both an X-ray and an MRI scan. In this sense, most articles that claim to evaluate the CSA do not consider this latter exam and, therefore, can access a larger number of subjects [49]. Additionally, we did not consider the clinical history of the patients, including the temporality of pain, clinical signs, family history, comorbidities, or any other pertinent questions that could enhance our research, such as psychosomatic factors [50]. Secondly, because the study framed its objective in a descriptive cross-sectional design, we did not consider a control group without alterations to the glenohumeral joint. Future contributions could lead to evaluating a comparison with similar samples, both with and without the diseases of interest, to determine the behavior of the described angle. Thirdly, it would be interesting to evaluate populations that obey different conditions. For example, athletes, sedentary individuals, labor-intensive activities of the upper limb, and even a younger population can see the temporal progress of the angle behavior over time according to demand [38]. Finally, it is essential to understand that each patient is unique, that the CSA is a sum of multidimensional factors that must be considered at the time of measurement, and that its follow-up over time responds to different biological and mechanical requirements.

## 5. Conclusions

The findings obtained in this investigation highlight the importance of considering CSA in RCI and GHOA injuries. It was observed that patients with IUGR were younger across all age ranges and had a higher CSA than patients with GHOA. Additionally, it was established that there were no differences between sexes, confirming previous international findings. We believe that the health status of the glenohumeral joint should be monitored continuously and that the use of this radiological tool can allow for a thorough record by the medical care team, anticipating symptomatic manifestations. New studies and radiological measurements in a broader population are necessary to continue validating and expanding knowledge in the musculoskeletal area.

## Figures and Tables

**Table 1:** Clinical variables by GHOA and RCI.

Variable		GHOA (n = 29) (mean, SD)/(%)		RCI (n = 30) (mean, SD)/(%)		*p*-Value (<0.05 *)
Age		72.9 ± 10.8	(43–96)	60.8 ± 7.6	(44–78)	0.001 *
Sex	Male Female	10 19	(34.5%) (65.5%)	16 14	(53.3%) (46.7%)	0.116
Dominant side	Right Left	19 10	(65.5%) (34.5%)	22 8	(73.3%) (26.7%	0.356
Injury side	Right Left Bilateral	11 16 2	(37.9%) (65.2%) (6.9%)	22 7 1	(73.3%) (13.3%) (3.3%)	0.015 *

*, statistical significance; GHOA, glenohumeral osteoarthritis; RCI, rotator cuff injury.

**Table 2:** CSA according to pathology.

Age	GHOA	RCI	*p*-Value (<0.05 *)
40–54	24.9	36.4	0.05 *
55–69	26.5	35.4	0.001 *
70–84	30.3	35.6	0.017 *
85–100	27.6	-	-

*, statistical significance; GHOA, glenohumeral osteoarthritis; RCI, rotator cuff injury.

## Data Availability

The raw data supporting the conclusions of this article will be made available by the authors without undue reservation.
